# Increase in burnout among physicians and associated factors in the period before and during the COVID-19 pandemic

**DOI:** 10.47626/1679-4435-2025-1442

**Published:** 2025-07-23

**Authors:** Renilda Martins Prestes, Luciano Ferreira Drager, Silvia Gonçalves Conway, Almir Ribeiro Tavares Junior, Márcia Assis, Andrea Frota Bacelar Rego, Claudia Roberta de Castro Moreno

**Affiliations:** 1 Department of Public Health, School of Public Health, University of São Paulo (USP), São Paulo, SP, Brazil; 2 Department of Pulmonology, Heart Institute (InCor), USP, São Paulo, SP, Brazil; 3 Hypertension Unit, InCor, USP, São Paulo, SP, Brazil; 4 Department of Psychology, Stress Research Institute, Stockholm University, Stockholm, Sweden

**Keywords:** Occupational health, public health, burnout, professional, COVID-19., saúde ocupacional, saúde pública, esgotamento profissional, covid-19.

## Abstract

**Introduction:**

Studies have demonstrated that individual and organizational factors contribute to burnout in health professionals. The COVID-19 pandemic increased demand for health care services, leading to work overload among health professionals, particularly physicians.

**Objectives:**

To determine the prevalence of burnout and identify possible individual, organizational, and sleep-related factors associated with burnout among physicians, before and during the COVID-19 pandemic.

**Methods:**

We conducted a cross-sectional study on a subsample of 2,639 physicians extracted from a secondary database with health professionals from all regions of Brazil, between May and June 2020. Descriptive and inferential statistical analyses were performed. Burnout was assessed via the emotional exhaustion dimension.

**Results:**

Data for 2,374 (90.3%) physicians were analyzed. The prevalence of burnout before the pandemic was 18.9%, increasing to 31.3% during the pandemic. The factors predicting burnout during the pandemic included age 25-39 years (odds ratio = 2.76; 95%CI 1.94-3.92), female sex (odds ratio = 1.67; 95%CI 1.34-2.08), working on the front line (odds ratio = 1.62; 95%CI 1.30-2.02), poor sleep quality and quantity (odds ratio = 6.39; 95%CI 4.99-8.17), and not working from home (odds ratio = 1.31; 95%CI 1.08-1.60).

**Conclusions:**

Compared to the work routine before the pandemic, there was a marked increase in the prevalence of burnout among physicians during the pandemic. Independent factors associated with this increase were young adult age, female sex, frontline work, poor sleep quality and quantity, and traditional on-site work.

## Abstract (pt)

**Introdução:**

Estudos demonstram que fatores individuais e organizacionais contribuem para o desenvolvimento de *burnout* em profissionais de saúde. A pandemia de covid-19 acentuou a demanda de atendimento nos serviços de saúde, gerando maior sobrecarga de trabalho, especialmente entre os médicos.

**Objetivos:**

Estimar a prevalência de *burnout* e identificar possíveis fatores individuais, organizacionais e distúrbios do sono associados ao *burnout* em médicos, antes e durante a pandemia de covid-19.

**Métodos:**

Estudo transversal realizado com uma subamostra de 2.639 médicos extraída de um banco de dados secundário com profissionais de saúde de todas as regiões do Brasil, entre maio e junho de 2020. Foram realizadas análises descritivas e testes de estatística inferencial. O *burnout* foi estimado a partir da análise do componente de esgotamento emocional.

**Resultados:**

Foram analisados dados de 2.374 (90,3%) médicos. A prevalência de *burnout* antes da pandemia foi de 18,9%, aumentando para 31,3% durante a pandemia. Os fatores preditores de *burnout* na pandemia foram faixa etária de 25 a 39 anos (*odds ratio* = 2,76; IC95% 1,94-3,92), sexo feminino (*odds ratio* = 1,67; IC95% 1,34-2,08), atuação na linha de frente (*odds ratio* = 1,62; IC95% 1,30-2,02), sono de menor quantidade e pior qualidade (*odds ratio* = 6,39; IC95% 4,99-8,17) e não trabalhar em *home office* (*odds ratio* = 1,31; IC95% 1,08-1,60).

**Conclusões:**

Em comparação ao período anterior à pandemia, observou-se um aumento expressivo na prevalência de *burnout* entre médicos durante a pandemia. Os fatores independentes associados a esse aumento foram faixa etária jovem adulto, sexo feminino, trabalho na linha de frente, sono insuficiente e de má qualidade e trabalho presencial.

## INTRODUCTION

Physicians work shifts that, due to sleep restriction, can lead to increased errors, work-related sharps injuries, and motor vehicle acidents.^^1^^ The nature of 24-hour medical shifts, along with the circadian misalignment experienced by shift workers, tends to worsen these professionals’ sleep problems. Nevertheless, the topic remains underexplored in literature.^^2^^

Physician well-being is considered an indicator of health system quality, with professional burnout described as a public health crisis.^^3^^ Burnout has received particular attention during the pandemic, with authors identifying physicians as the health professionals most susceptible to developing it due to the very nature of their work - providing patient care.^^4^^

Studies have shown that sociodemographic factors, both individual and organizational, may contribute to the development of burnout among physicians. Among individual factors, female sex has been most frequently associated with burnout in these professionals.^^5^^ Age is also considered an individual factor significantly associated with burnout.^^1^^ Regarding the work environment, serving on the front lines in cities remote from the pandemic epicenter appears to be a protective factor against mental health impairment.^^6^^

The high prevalence of burnout in this population - 54.5% among American pediatric residents in 2017^^7^^ and 86% among Italian physicians during the pandemic^^8^^ - underscores the importance of investigating this issue. In scientific research, studying the prevalence of burnout among physicians appears to broaden understanding of risk factors for occupational exhaustion. The results of the present study, based on the thesis “Prevalence and factors associated with burnout in physicians during the COVID-19 pandemic,” are relevant for planning measures to protect physicians’ health both in their routine practice and in future large-scale health emergencies.

## METHODS

### CHARACTERISTICS OF THE DATABASE OF A BRAZILIAN CROSS-SECTIONAL STUDY INVOLVING HEALTH CARE PROFESSIONALS DURING THE COVID-19 PANDEMIC

**Figure 1:**
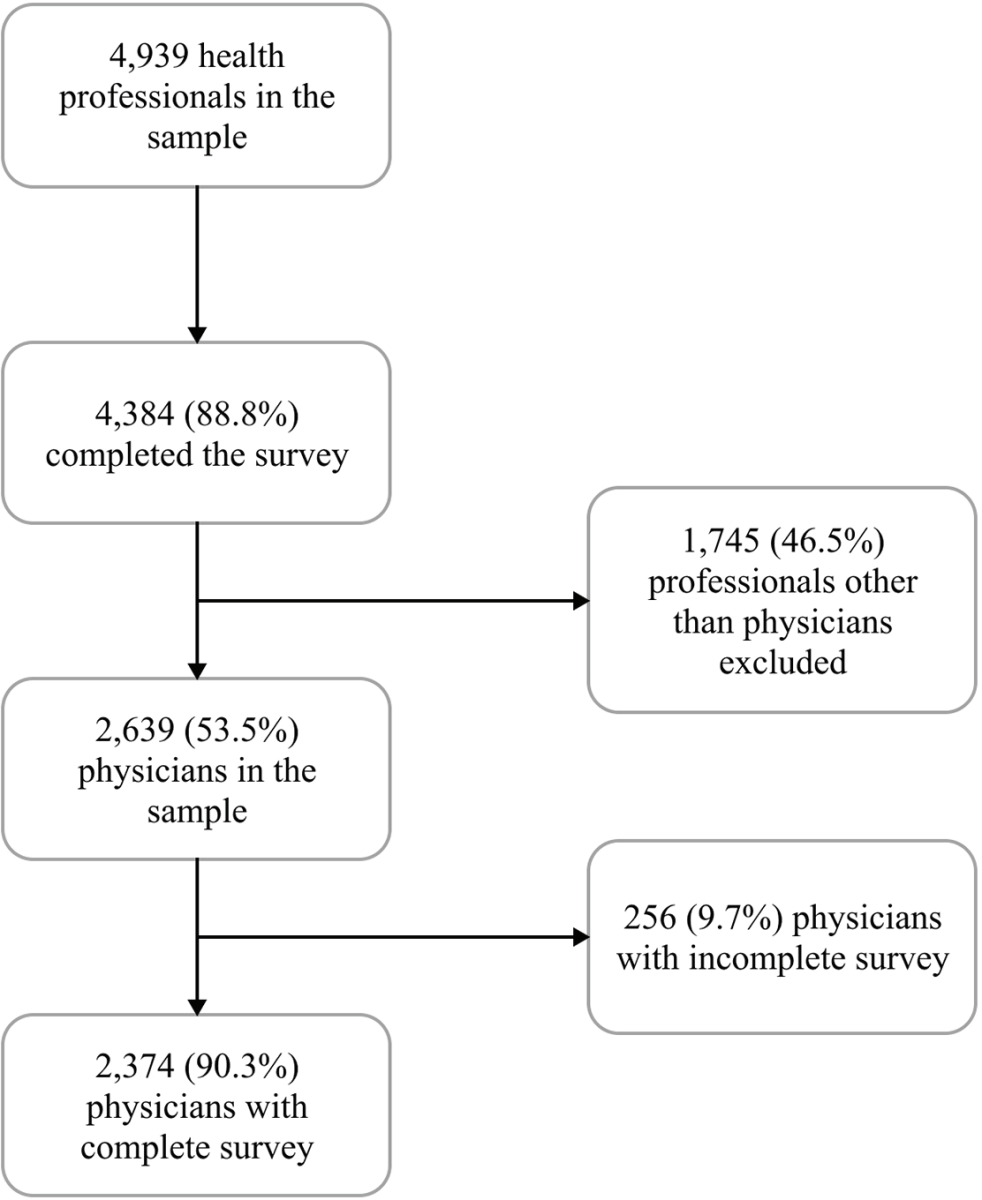
Sample size and participant selection.

The database used was developed by sleep specialists between May 28 and June 28, 2020. The initial sample comprised 4,939 health care professionals working in hospitals across Brazil’s five regions, of whom 2,639 were physicians (53.4% of the initial sample).^^9^^ Of these, 2,374 (90.3% of the total physicians) fully completed the questionnaire, constituting the final sample of this study, as shown in Figure 1.

The variables analyzed in this study included sex, age, work organization, working hours, deployment to non-frontline settings, remote work, and sleep disorders. Burnout was assessed using an adapted, single-item scale - previously used and validated - to measure perceived work exhaustion.^^10^^

Sex was categorized as female or male, and age was expressed in years. Regarding the workplace setting, participants could select more than one sector: ward, intensive care unit (ICU)/semi-intensive care unit (SICU), office/clinic, sleep laboratory, surgical center, pharmacy, and administrative area. Working hours were reported as the current weekly schedule and the schedule before the pandemic. Regarding remote work, participants indicated whether they worked remotely, even partially, with “yes” or “no” responses.

The sleep variable was assessed regarding sleepiness, insomnia, and sleep quality and quantity. Sleep quality and quantity were assessed based on whether they were maintained, worsened, or improved. Current and pre-pandemic sleep duration were evaluated according to the average number of hours slept per night. Insomnia was assessed using a specific item that evaluated weekly frequency, ranging from 1 to 5 or more times per week.^^11^^

### PRESENTATION OF THE SELF-DEFINED BURNOUT IDENTIFICATION INSTRUMENT

Burnout assessment in research is generally performed using self-administered questionnaires,^^12^^ with the Maslach Burnout Inventory (MBI), developed in 1981, being the oldest and regarded as the reference standard for this type of analysis.^^13^^ The single-item instrument is a validated tool comprising a single question with responses rated on a five-point Likert scale, intended to identify self-defined burnout.^^13^^ Among Japanese physicians, the single-item instrument demonstrated a sensitivity of 53.8% and a specificity of 88.2% for identifying burnout.^^13^^ Considering the sample size and the challenges of administering an extensive questionnaire, the present study employed the single-item measure to detect burnout.

### STATISTICAL ANALYSES

Initially, the loss rate was calculated by determining the proportion of respondents with incomplete questionnaires relative to the total number of participants. Comparisons were then made between included participants and losses according to sex, age, and average nightly sleep duration before and during the pandemic. For this purpose, the chi-square test was used for qualitative variables and the Mann-Whitney test for nonparametric quantitative variables. Subsequently, data descriptive analysis was performed using means, standard deviations (SD), and minimum and maximum values for quantitative variables, and proportions for qualitative variables.

For quantitative variables, the Shapiro-Francia test was applied to assess adherence to normal distribution, which determined the choice of statistical tests to be used. The outcome variable, “presence of burnout,” was defined by an affirmative response regarding perception of burnout symptoms. As the quantitative variables exhibited a nonparametric distribution, the Wilcoxon test (for comparing two related means) and the McNemar test (for comparing repeated measures of dichotomous variables) were used for repeated measures comparisons.

To identify factors associated with burnout, association analyses were initially performed using Pearson’s chi-square test. Subsequently, multiple logistic regression analysis was performed, including variables with theoretical relevance to burnout and those with p < 0.200 in the univariate analysis. Multiple analysis was performed using a forward stepwise method, with the p-value of each variable determining its order of entry into the multivariable model. The measure of association was the odds ratio (OR), and model fit was evaluated by the Hosmer-Lemeshow test. When necessary, variable categories were collapsed for multivariable analysis due to their frequency distributions.

The significance level for identifying associations was set at p < 0.050 in all analyses, and the 95%CI of the OR was also considered in the logistic regression. Data were collected via Google Forms. Subsequently, data were exported to Excel for score calculation and then to Stata 14 for consistency checks, variable recoding, and statistical analyses.

## RESULTS

### LOSS ANALYSES

Among health care professionals from Brazil’s five regions who responded to the questionnaire during the COVID-19 pandemic, 2,639 were physicians. From this group, 2,374 (90.3%) fully completed the questionnaire and were included in the present study. The remaining 256 physicians had incomplete questionnaires, resulting in a 9.7% loss rate.

### CHARACTERISTICS OF THE STUDY POPULATION

#### Demographic and occupational characteristics

Regarding sex, a greater proportion of participants were female (67.7%). The most frequent age groups were 30-39 and 40-49 years, at 27.1% and 27.2%, respectively. The mean age was 45.7 years (SD = 11.9 years), ranging from 23.0 to 80.0 years, with a median age of 45.0 years. The most common workplace setting was office/clinic (71.4%), followed by hospital ward (26.9%) and ICU/SICU (22.9%).

Before the pandemic, 50% of physicians worked up to 40 hours per week; at data collection, it rose to 67.1%, with fewer physicians in the higher workload categories. Mean weekly working hours decreased from 46.1 hours (median = 41.0 hours, SD = 17.8 hours) before the pandemic to 37.0 hours (median = 36.0 hours, SD = 21.1 hours) during data collection. This difference was statistically significant (p < 0.001 - Wilcoxon signed-rank test for repeated measures).

### SLEEP CHARACTERISTICS

Regarding sleep quality compared to the pre-pandemic period, 36.4% reported no change, while 58.0% reported worsening. Regarding sleep quantity, 36.7% reported no change and 43.8% reported a decrease. Regarding sleepiness degree, 24.9% of physicians reported feeling active, alert, and well-disposed; 47.4% stated they could concentrate but were not at peak alertness; 11.7% described themselves as relaxed, awake, and responsive yet not fully alert; and 16.0% reported experiencing some degree of sleepiness. In terms of perceived sleepiness during the pre-pandemic period, 24.2% reported more sleepiness than now, whereas 41.2% reported experiencing less.

Before the pandemic, 90.5% of physicians slept ≥ 6 hours per night; this proportion fell to 75.3% at data collection. The mean sleep duration per night before the pandemic was 6 hours 48 minutes (median = 7.0 hours, SD = 1.0), decreasing to 6 hours 36 minutes (median = 6.5 hours, SD = 1.5) at data collection. This difference was statistically significant (p < 0.001 - Wilcoxon signed-rank test for repeated measures). Regarding insomnia, 19% reported episodes once per week, 30.2% two to four times per week, and 9.2% five or more times per week. In total, 1,387 (58.4%) physicians reported insomnia.

### PRESENCE OF BURNOUT

**Figure 2:**
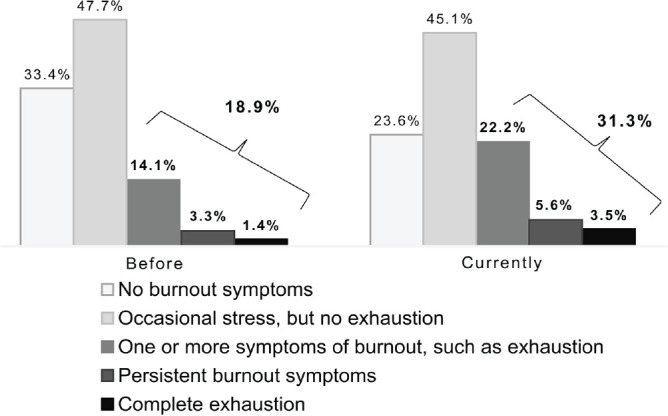
Perceived presence of burnout among the study’s physician population during the COVID-19 pandemic, Brazil, 2023.

Figure 2 shows the perceived presence of burnout among the study’s physician population during the COVID-19 pandemic. Burnout prevalence was 18.9% before the pandemic, based on questionnaire responses related to exhaustion, and rose to 31.3% at data collection.

Table 1 presents perceived burnout before and during the COVID-19 pandemic. Compared with the pre-pandemic period, the odds of experiencing burnout during the pandemic were 3.279 times higher.

**Table 1:** Perceived burnout in physicians before and during the COVID-19 pandemic, Brazil, 2023

Currently	Pre-pandemic				Total		OR (95%CI)^*^
	Burnout (n)	%	No burnout (n)	%	n	%	
Burnout	319	13.4	423	17.8	742	31,3	3.279 (2.686-4.025)
No burnout	129	5.4	1,503	63.3	1,632	68.7	
Total	448	18.8	1,926	81.1	2,374	100.0	

OR = odds ratio.

* McNemar test for repeated measures.

### ANALYSIS OF FACTORS ASSOCIATED WITH THE PRESENCE OF BURNOUT

#### Univariate analysis

Table 2 presents the results of association tests between demographic and occupational characteristics and perceived burnout at data collection. Sex was significantly associated with burnout (p < 0.001), with a higher prevalence among females (35.8%) than males (21.8%). Age group also showed a significant association (p < 0.001), with burnout prevalence decreasing as age increased. The highest prevalences were observed in the 25-29 years (45.3%) and 30-39 years (40.6%) age groups.

**Table 2:** Distribution of the study population by demographic and occupational characteristics and presence of burnout currently among physicians who worked during the COVID-19 pandemic, Brazil, 2023

Characteristics	Presence of burnout currently						p-value^*^
	No		Yes		Total		
	n	%	n	%	n	%	
Sex							
Male	599	78.2	167	21.8	766	100.0	< 0.001
Female	1,033	64.2	575	35.8	1,608	100.0	
Age group (years)							
25-29	104	54.7	86	45.3	190	100.0	< 0.001
30-39	382	59.4	261	40.6	643	100.0	
40-49	446	69.0	200	31.0	646	100.0	
50-59	417	74.7	141	25.3	558	100.0	
60-69	220	82.4	47	17.6	267	100.0	
70-80	63	90.0	7	10.0	70	100.0	
Work setting - ward							
No	1,243	71.6	492	28.4	1,735	100.0	< 0.001
Yes	389	60.9	250	39.1	639	100.0	
Work setting - ICU/SICU							
No	1,330	72.6	501	27.4	1,831	100.0	< 0.001
Yes	302	55.6	241	44.4	543	100.0	
Work setting - office/clinic							
No	435	64.0	245	36.0	680	100.0	0.001
Yes	1,197	70.7	497	29.3	1,694	100.0	
Work setting - sleep laboratory							
No	1,580	68.3	734	31.7	2,314	100.0	0.002
Yes	52	86.7	8	13.3	60	100.0	
Work setting - surgical center							
No	1,277	67.1	625	32.9	1,902	100.0	0.001
Yes	355	75.2	117	24.8	472	100.0	
Work setting - pharmacy							
No	1,631	68.7	742	31.3	2,373	100.0	0.500
Yes	1	100.0	0	0.0	1	100.0	
Work setting - administrative area							
No	1,486	68.7	678	31.3	2,164	100.0	0.799
Yes	146	69.5	64	30.5	210	100.0	
Number of work settings							
1	985	72.0	384	28.0	1,369	100.0	0.001
2	449	63.2	261	36.8	710	100.0	
3-5	176	66.9	87	33.1	263	100.0	
None	22	68.8	10	31.3	32	100.0	
Weekly working hours before pandemic							
0-40	878	74.0	309	26.0	1,187	100.0	< 0.001
41-50	303	72.3	116	27.7	419	100.0	
51-60	291	58.0	211	42.0	502	100.0	
61-160	160	60.2	106	39.8	266	100.0	
Current weekly working hours†							
0-40	1,189	74.6	404	25.4	1,593	100.0	< 0.001
41-50	168	63.2	98	36.8	266	100.0	
51-60	148	52.1	136	47.9	284	100.0	
61-160	127	55.0	102	44.5	229	100.0	
Remote work (even if partial)							
Yes	766	72.5	290	27.5	1,056	100.0	< 0.001
No	866	65.7	452	34.3	1,318	100.0	
Treated/treats COVID-19 patients							
Yes	1,014	65.0	547	35.0	1,561	100.0	< 0.001
No	618	76.0	195	24.0	813	100.0	
Total	1,632	68.7	742	31.3	2,374	100.0	

ICU = intensive care unit; SICU = semi-intensive care unit.

* Chi-square test.

† Two subjects excluded due to inconsistent responses.

Work setting was significantly associated with burnout (p < 0.001), with a higher prevalence among physicians working in the ICU/SICU (44.4%) compared to other settings (27.4%).

Regarding weekly working hours, statistically significant differences were observed (p < 0.001) both in the pre-pandemic period and during data collection. Before the pandemic, physicians working more than 50 or more than 60 hours per week (approximately 40% of physicians) exhibited the highest burnout prevalence. During data collection, about 50% of physicians working 51-60 hours per week reported burnout. Burnout was significantly higher among those working from home (27.5%) and those on-site (34.3%) (p < 0.001).

Table 3 presents the results of association tests between sleep characteristics and perceived burnout at data collection. Worsened sleep quality was significantly linked to burnout (p < 0.001), with 44% of physicians reporting poorer quality experiencing burnout. Similarly, 46.3% of those with reduced sleep quantity had burnout (p < 0.001).

**Table 3:** Distribution of the study population by sleep characteristics and presence of burnout currently among frontline physicians during the COVID-19 pandemic, Brazil, 2023

Characteristics	Presence of burnout currently						p-value^*^
	No		Yes		Total		
	n	%	n	%	n	%	
Overall sleep quality							
Same	738	85.4	126	14.6	864	100.0	< 0.001
Worsened	772	56.0	606	44.0	1,378	100.0	
Improved	122	92.4	10	7.6	132	100.0	
Overall sleep quantity							
Same	697	79.9	175	20.1	872	100.0	< 0.001
Worsened	559	53.8	481	46.3	1,040	100.0	
Improved	376	81.4	86	18.6	462	100.0	
Sleepiness degree							
Feels active, alert, and well-disposed	556	94.1	35	5.9	591	100.0	< 0.001
Can concentrate but is not at peak alertness	777	69.1	348	30.9	1,125	100.0	
Relaxed, awake, and responsive but not fully alert	155	55.8	123	44.2	278	100.0	
Sleepy but awake	87	41.6	122	58.4	209	100.0	
Very sleepy, almost dozing, no interest in staying awake, slow thinking	13	20.6	50	79.4	63	100.0	
Prefers lying down, fighting sleep	37	46.3	43	53.8	80	100.0	
Losing the fight against sleep, on the verge of dozing	7	25.0	21	75.0	28	100.0	
Sleepiness relative to the pre-pandemic period							
Less sleepy than currently	554	56.6	424	43.4	978	100.0	< 0.001
Sleepier than currently	428	74.6	146	25.4	574	100.0	
No sleepiness	650	79.1	172	20.9	822	100.0	
Hours of sleep per night before the pandemic							
8 or more	362	61.0	231	39.0	593	100.0	< 0.001
Less than 8 and more than 6	1,110	71.4	445	28.6	1,555	100.0	
Less than 6	160	70.8	66	29.2	226	100.0	
Current hours of sleep per night							
8 or more	475	74.9	159	25.1	634	100.0	< 0.001
Less than 8 and more than 6	850	73.7	304	26.3	1,154	100.0	
Less than 6	307	52.4	279	47.6	586	100.0	
Insomnia episodes							
No	798	81.8	178	18.2	976	100.0	< 0.001
Yes, but unchanged	373	79.7	95	20.3	468	100.0	
Yes, worsened	461	49.6	469	50.4	930	100.0	
Insomnia frequency							
No insomnia	798	81.8	178	18.2	976	100.0	< 0.001
Once per week	344	76.3	107	23.7	451	100.0	
2-4 times per week	393	54.8	324	45.2	717	100.0	
More than 5 times per week	92	42.0	127	58.0	219	100.0	
Total	1,632	68.7	742	31.3	2,374	100.0	

These questions compare the pre-pandemic period with the current situation.

* Chi-square test.

Regarding sleepiness, lower burnout rates (below 45%) were observed among physicians reporting lower levels of sleepiness, with statistically significant differences (p < 0.001). There was also a significant association for perceived sleepiness compared to the pre-pandemic period (p < 0.001). The highest burnout rate (43.4%) was seen in participants who reported feeling less sleepy during the pandemic than during data collection period.

Pre-pandemic sleep quantity correlated with burnout (p < 0.001): physicians sleeping ≥ 8 hours nightly showed the highest burnout rate (39%). During data collection, sleep duration also correlated with burnout (p < 0.001): physicians sleeping < 6 hours nightly had the highest rate (47.6%).

Insomnia was significantly associated with burnout (p < 0.001). Physicians with pre-existing insomnia who reported worsening during data collection had the highest burnout rate (50.4%). Burnout prevalence was 45.2% among those with insomnia two to four times per week and 58.0% among those with insomnia five or more times per week (p < 0.001).

#### Multiple logistic regression analysis

Table 4 presents the results of the multiple logistic regression analysis. Variables independently associated with burnout at data collection were age group, sex, working in the ICU/SICU, and sleep quality and quantity.

**Table 4:** Multiple logistic regression analysis of factors associated with the presence of burnout in the study population of physicians before and during a period of the COVID-19 pandemic, Brazil, 2023

Variable	aOR	95%CI (aOR)		p-value
		Inferior	Superior	
Age groups (years)				
60-80	1.00			
40-59	1.68	1.20	2.37	0.003
25-39	2.76	1.94	3.92	< 0.001
Sex				
Male	1.00			
Female	1.67	1.34	2.08	< 0.001
Works in ICU/SICU				
No	1.00			
Yes	1.62	1.30	2.02	< 0.001
Sleep quality and duration				
Quality and duration maintained or improved	1.00			
Duration worsened	1.96	1.22	3.15	0.005
Quality worsened	3.20	2.40	4.26	< 0.001
Quality and duration worsened	6.39	4.99	8.17	< 0.001
Remote work (even if partial)				
Yes	1.00			
No	1.31	1.08	1.60	0.006
Treated/treats COVID-19 patients				
No	1.00			
Yes	1.10	0.80	1.51	0.543

Model adjustment variable: “having treated or treating COVID-19 patients”.

aOR = adjusted odds ratio; ICU = intensive care unit; SICU = semi-intensive care unit.

Compared with professionals in the oldest age group (60-80 years), participants aged 40-59 years had 1.68-fold higher odds of burnout (95%CI 1.20-2.37), while those aged 25-39 years had 2.76-fold higher odds (95%CI 1.94-3.92). Female sex was associated with 1.67-fold higher odds of burnout (95%CI 1.34-2.08) compared with male sex. Participants working in the ICU/SICU had 1.62-fold higher odds of burnout (95%CI 1.30-2.02) than those not working in these units.

Interaction analysis between sleep quality and duration showed that, compared with physicians reporting simultaneous improvement in both domains, those with worsened duration had OR 1.96 (95%CI 1.22-3.15) for burnout; those with worsened quality had OR 3.20 (95%CI 2.40-4.26); and those with worsened quality and duration had OR 6.39 (95%CI 4.99-8.17). The model was adjusted for the variable “having treated or treating COVID-19 patients.” Model fit, assessed by the Hosmer-Lemeshow test (χ^^2^^ = 12.74; p = 0.121), indicated good fit.

## DISCUSSION

The prevalence of burnout among physicians in this study was 18.9% before the COVID-19 pandemic, increasing to 31.3% during the pandemic. Literature reports variable burnout rates. In a study of Chinese primary care physicians, a prevalence of 35% was found when any component of burnout was considered, and 24.8% when only emotional exhaustion was evaluated.^^14^^

Frontline work with COVID-19 patients was associated with a statistically significant increase in burnout, with 3.279 times higher odds of developing the syndrome during the pandemic compared to the pre-pandemic period. This exceeds international findings, which report odds ratios of 1.92 in East Asia and the Pacific, 1.19 in Europe and Central Asia, 1.40 in Latin America and the Caribbean, 1.28 in the Middle East and North Africa, 1.78 in South Asia, and 1.72 in Sub-Saharan Africa.^^15^^ One possible explanation for the higher odds of burnout observed in this study compared to other countries is the variation in health system protocols adopted in each setting.

Analysis also indicated that female physicians had 1.67 times higher odds of developing burnout compared with male physicians. Similar results were observed in a study of Ibero-American pediatric urologists, in which females had 3.26 times higher odds of burnout than males.^^16^^

Age also demonstrated a significant association with burnout in this study. For analysis and comparison with other research, age was grouped into ranges, with higher burnout prevalence observed in the younger brackets. Age over 59 years may act as a protective factor against burnout, possibly because younger physicians face greater work pressure and professional responsibilities.

Working hours warrant attention when assessing physician burnout, especially during a pandemic given these professionals’ critical role in caring for infected patients. In present study’s final model, weekly hours were not associated with burnout. This may reflect a non-linear relationship between longer hours and burnout. Indeed, physicians working over 60 hours per week had a lower burnout rate than those working 51-60 hours.

In this study, burnout prevalence was higher among physicians working in ICU/SICU compared to other sectors, with 1.62-fold increased odds of burnout in these units. Higher prevalence may be due to the greater clinical severity of patients treated in these frontline settings relative to the other sectors examined.

In this study, we found a significant association between burnout and frontline work during the pandemic. However, literature reports opposite results. In a Chinese study, for example, burnout prevalence was lower on the front line (13%) than in the ward (39%), when considering the components of emotional exhaustion and depersonalization.^^17^^ One possible explanation for this is that access to more up-to-date and robust information about the disease among frontline physicians may have contributed to a greater sense of security in these professionals.

The COVID-19 pandemic’s high patient load and the need for rapid clinical management drove the widespread adoption of telemedicine, previously confined to health care services and little used by the general population. Although literature provides robust data on telemedicine within health systems, studies on remote work are scarce. Our results reveal a statistically significant association between burnout and both physicians working remotely and those working on-site. Further research is needed to explain these findings.

During the pandemic, physicians have a 2-fold higher risk of developing burnout compared with the general population, owing to the nature of their work. Other factors that may contribute to burnout include sleep disorders.^^18^^ In this study, both sleep quality and quantity were significantly associated with burnout: physicians reporting worsened quality and/or quantity had the highest burnout prevalence. Nightly sleep quantity showed an association with burnout both before the pandemic and at data collection.

Nightly sleep under 6 hours was associated with higher burnout. Physicians with worsened sleep quantity had 1.96-fold greater odds of burnout than those reporting improved sleep quality and quantity. Frontline physicians with declines in both sleep quality and quantity had 6.39-fold greater odds of burnout than colleagues without such declines. Elevated sleepiness also correlated significantly with burnout.

In a study of 1,306 physicians from multiple hospitals in Hubei, China, 71.7% reported poor sleep quality and nearly half (45.5%) experienced insomnia. A significant association was also found between frontline work and sleep quality.^^19^^ These results mirror our findings: frontline and female physicians had 1.67-fold higher odds of burnout, and those with poor sleep quality had 3.2-fold higher odds of burnout compared with physicians without these impairments.

Insomnia was significantly associated with burnout: the highest rates occurred among physicians with pre-existing insomnia who reported worsening during the pandemic. Burnout was most common among those with more weekly insomnia episodes, a statistically significant difference from other groups. However, insomnia was excluded from the final model, possibly due to the lack of a consistent linear pattern. For example, among physicians with burnout, 20.3% reported no change in insomnia during the pandemic, whereas 50.4% reported worsening.

One should consider that incomplete questionnaire responses resulted in approximately 10% sample loss. However, according to the statistical analyses performed, it is unlikely that this loss influenced the results, since data from incomplete items were retained rather than excluding participants with partial responses entirely. Despite the sample’s robustness, the possibility of healthy volunteer bias, which may have reduced interest in participation and consequently the sample size, should be acknowledged.

In summary, burnout prevalence among physicians, assessed via the emotional exhaustion component, increased by 12.4% during the pandemic. Female sex, younger age, frontline COVID-19 duties, and insufficient sleep quality and quantity were associated with higher odds of burnout in this study.

## CONCLUSIONS

The results of this study show that during the pandemic, the sociodemographic and sleep-related factors associated with burnout in physicians were younger adult age, female sex, frontline COVID-19 care, and insufficient sleep quality and quantity. Given the diversity of these factors, measures to prevent burnout among physicians may include organizational and individual management of physicians’ work routines, as well as in future public health emergencies - more assertive actions should be implemented by health system managers.

### IMPLICATIONS OF THE STUDY

This study’s contributions lie in its strong potential to generate knowledge for understanding physician burnout and identifying factors that contribute to it, so they can be addressed. With this insight, it should be possible to support the organizational management of these professionals by implementing preventive measures against exhaustion in clinical practice and in future large-scale public health crises.

### ETHICAL DECLARATIONS

#### Ethical aspect

The database was reviewed and approved by the Research Ethics Committee of the Hospital das Clínicas, Faculty of Medicine, University of São Paulo, under Certificate of Presentation for Ethical Approval (Certificado de Apresentação de Apreciação Ética, CAAE) number 31750920.9.0000.0068. The use of this secondary database, developed by sleep specialists from a cross-sectional study, was exempted from the requirement for an Informed Consent Form by the Ethics Committee. It should be noted that the present study used data provided by the researchers of the approved study, and the investigator responsible for the analysis presented here did not have access to participants’ personal data.
